# Supplementary material for: Occurrence and Identification of Yeasts in Production of White-Brined Cheese
Source: Microorganisms. 2022 May 24;10(6):1079. doi: 10.3390/microorganisms10061079 (PMC9228510; doi:10.3390/microorganisms10061079)
Supplement: Supplementary file 1 [file microorganisms-10-01079-s001.zip › microorganisms-1741097-supplementary.pdf]

Table S1. Fermentation and assimilation of carbohydrates by *Kluyveromyces* spp. isolates from production line of white-brined cheese

Isolate Code	Fermentation of carbohydrates [a]						Assimilation of carbohydrates [b]					
	Glucose	Galactos	Sucrose	Maltose	Lactose	Raffinos	Glucose	Galactos	Sucrose	Maltose	Lactose	Raffinos
T2-5	+	+	+	-	+	w	+	+	+	+	+	+
T2-10	+	+	+	-	+	w	+	+	+	+	+	+
T2-22	+	+	+	-	+	w	+	+	+	+	+	+
T2-27	+	+	+	-	+	w	+	+	+	+	+	+
T2-28	+	+	+	-	+	w	+	+	+	+	+	+
T2-29	+	+	+	-	+	w	+	+	+	+	+	+
Kluyveromyces lactis CBS 845	+	+	+	+	+	w	+	+	+	+	+	+
Kluyveromyces marxianus CBS 1553	+	+	+	-	+	w	+	+	+	+	+	+

[a] Results are given as + (strongly positive, Durham tube filled with air within 7 days), l (delayed positive, Durham tube filled quickly after 7 days), s (slow positive, Durham tube filled slowly after 7 days), w (weakly positive, under 1/3 of the Durham tube is filled with air), and – (negative, no production of air).

[b] Results are given as + (positive, 2+ or 3+ on Wickerhams card after 1 week), l (delayed positive, 2+ or 3+ on Wickerhams card after 2 weeks), s (slow positive, 2+ or 3+ on Wickerhams card in up to 4 weeks), w (weak positive, 1+ on Wickerhams card), and – (negative, no growth).
